# Network pharmacology analysis reveals neuroprotection of *Gynostemma pentaphyllum* (Thunb.) Makino in Alzheimer’ disease

**DOI:** 10.1186/s12906-022-03534-z

**Published:** 2022-03-07

**Authors:** Jiahao Wang, Jiamiao Shi, Ning Jia, Qinru Sun

**Affiliations:** 1 Xi’an Jiaotong University Health Science Center , Xi’an, Shaanxi 710061 People’s Republic of China; 2 Department of Human Anatomy, Histology and Embryology, School of Basic Medical Sciences, Xi’an Jiaotong University Health Science Center, No. 76, West Yanta Road, Xi’an, Shaanxi 710061 People’s Republic of China; 3 Institute of Forensic Medicine, Xi’an Jiaotong University Health Science Center, No. 76, West Yanta Road, Xi’an, Shaanxi 710061 People’s Republic of China

**Keywords:** AD pathology, Molecular mechanisms, Alzheimer’s disease, *Gynostemma pentaphyllum* (Thunb.) Makino, Network pharmacology

## Abstract

**Background:**

Alzheimer’s disease (AD) is one of the most common neurodegenerative disorders in the world, but still lack of effective drug treatment. *Gynostemma Pentaphyllum* (Thunb.) Makino (GpM), a Chinese medicinal herb, plays important roles in anti-inflammation, anti-oxidative stress and anti-tumor, which has been reported to ameliorate cognitive impairment of AD. However, the neuroprotective mechanism of GpM remains unclear. This study aims to investigate the targets and possible signaling pathways of GpM in the treatment of AD.

**Methods:**

Active compounds of GpM and their putative target proteins were selected from Traditional Chinese Medicine Systems Pharmacology (TCMSP) Database and Analysis Platform. AD-associated targets were identified from GeneCards, the Online Mendelian Inheritance in Man (OMIM) database and the Therapeutic Target Database (TTD). The intersecting targets of GpM and AD were identified and Gene Ontology (GO), Kyoto Encyclopedia of Genes and Genomes (KEGG) analysis were carried out to analyze the mechanism of them. Compound-target-pathway (CTP) network and protein–protein interaction (PPI) network were constructed and analyzed to elucidate the correlation between compounds, proteins and pathways. Molecular docking was performed to further demonstrate the possibility of GpM for AD.

**Results:**

A total of 13 active compounds of GpM, 168 putative target proteins of compounds and 722 AD-associated targets were identified. Eighteen intersecting targets of GpM and AD were found and the epidermal growth factor receptor (EGFR), interleukin-1 beta (IL-1β), interleukin-6 (IL-6), nitric oxide synthase in endothelial (NOS3) and serum paraoxonase/arylesterase 1 (PON1) were selected as the primary targets of GpM in the treatment of AD. The neuroprotective effect of GPM was related to a variety of pathways, including amoebiasis, HIF-1 signaling pathway, cytokine-cytokine receptor interaction and so on.

**Conclusions:**

Our findings elucidate the active compounds, targets and pathways of GpM involved in effects of anti-AD. The novel mechanism of GpM against AD provides more treatment options for AD.

**Supplementary Information:**

The online version contains supplementary material available at 10.1186/s12906-022-03534-z.

## Background

Alzheimer’s disease (AD) is a neurodegenerative disease with progressive dementia which is characterized by neuronal loss, neuroinflammation and pronounced memory decline. Epidemiological findings show that the incidence rate of AD is increasing with age. Taking care of AD patients require a lot of time and financial resources, which brings huge burden and impact to society and family [1]. Current studies have demonstrated that extracellular accumulation of amyloid beta (Aβ) peptide [2], dysfunction of cholinergic system [3], neurofibrillary tangles (NFTs) of hyper-phosphorylated tau protein [4], and multiple genes including ABCA7, BIN1, CASS4, CD33, CD2AP, CELF1, CLU, CR1 and DSG2 are involved in the occurrence and progression of AD [5]. Substantial studies and clinical trials have demonstrated that it is difficult to develop one medicine for the treatment of AD, because the pathogenesis is very complex, including neurochemicals, amyloid and tau pathological processes, mitochondria, inflammatory pathways and neuroglia [6]. Chinese herbal medicine has a long history and plays a great role in the treatment of nervous system diseases. Its neuroprotective effect in AD has been confirmed by previous studies [7]. In a recent review, the authors believe that Chinese herbal medicine may be beneficial in improving cognitive function of AD patients. However, a largescale and multi-center research should be conducted to evaluate its benefits in the treatment of AD [8]. Therefore, the development of traditional Chinese medicine in the treatment of AD should be a long-term exploration.

*Gynostemma pentaphyllum* (Thunb.) Makino (GpM) is one of Chinese medicinal herbs, mainly distributed in northeast and southeast of Asia. Previous studies have shown that GpM has various effects, such as anti-inflammation [9], anti-oxidative stress [10], immune regulation [], anti-cancer [12], anti-aging [13] and prevention of cardiovascular diseases [14]. In recent years, GpM has been shown to alleviate brain lesion in chronic cerebral hypoperfusion [15] and process neuroprotection against 1-methyl-4-phenylpyridinium [16]. It is worth noting that GpM has been shown strong neuroprotective effects in the treatment of AD [17,18]. However, the neuroprotective mechanism of GpM in AD has not been fully studied.

In the present study, in order to investigate the potential pharmacological and molecular mechanisms of GpM in the treatment of AD, multiple databases and bioinformatics analysis were applied. The results showed that multiple pathways and targets were involved in the neuroprotection of GpM in the treatment of AD.

## Methods

### Screening for active compounds, putative target proteins and AD-associated genes

The active compounds of GpM and their putative target proteins were obtained from the Traditional Chinese Medicine Systems Pharmacology (TCMSP) Database and Analysis Platform (https://old.tcmsp-e.com/tcmsp.php) [19]. As a unique database containing a large number of herbs, active ingredients, and their targets, TCMSP contains pharmacokinetic properties of active compounds, such as oral bioavailability (OB), drug-likeness (DL) and so on. Oral bioavailability, as one of the most crucial pharmacokinetic parameters, shows the rate at which drugs enter the blood circulation. The greater the OB value of a compound, the more likely it is to become an effective drug. The drug-likeness (DL) represents the possibility of the compound becoming a drug. Due to poor pharmacological activity, most compounds in Traditional Chinese Medicine (TCM) cannot become effective drugs. OB ≥30% and DL ≥0.18 are considered as the criteria for screening clinical drugs [20]. The putative target proteins of active compounds were collected from TCMSP database, because the database contains the target proteins of each active compound. All information about the active compounds and putative target proteins of GpM was obtained from TCMSP database.

The AD-associated genes in this study were collected from 3 databases, which are GeneCards (http://www.genecards.org/) [21], Online Mendelian Inheritance in Man (OMIM) (https://omim.org/) [22] database and Therapeutic Target Database (TTD) (http://db.idrblab.net/ttd/) [23].

### Gene ontology (GO) analysis and Kyoto encyclopedia of genes and genomes (KEGG) pathway enrichment analysis

Eighteen overlap targets between 168 putative compound target proteins and 722 AD-associated genes were considered as therapeutic targets for AD. In order to elucidate the pathogenesis of AD and clarify the mechanism and function of GpM, 18 intersecting targets were analyzed by Metascape (http://metascape.org/) [24]. Metascape is a website integrating gene annotation and analysis resources with more than 40 databases, combined with GO analysis and KEGG pathway enrichment analysis. Results were considered significant at *P* < 0.01.

### Compound-target-pathway network establishment

The information of the components, targets and pathways was intuitively analyzed and shown a compound-target-pathway network by using Cytoscape 3.8.2 software (http://www.cytoscape.org/) [25].

### Protein-protein interaction (PPI) networks establishment

PPI data were obtained from the STRING database (https://string-db.org/) [26], and the result of PPI data was visualized by Cytoscape software.

### Molecule docking

By using Discovery Studio Client v19.1.0.18287, AutoDockTools-1.5.6 and PyMOL, the binding sites, capacities and interactions between compounds and proteins were analyzed [27–29]. The crystal structures of core targets were obtained from the Protein Data Bank (http://www.pdb.org/) [30, 31]. The 3D chemical structural formulas of quercetin were obtained from PubChem (https://pubchem.ncbi.nlm.nih.gov/) [32].

## Results

### Active compounds and putative target proteins

**Fig. 1:**
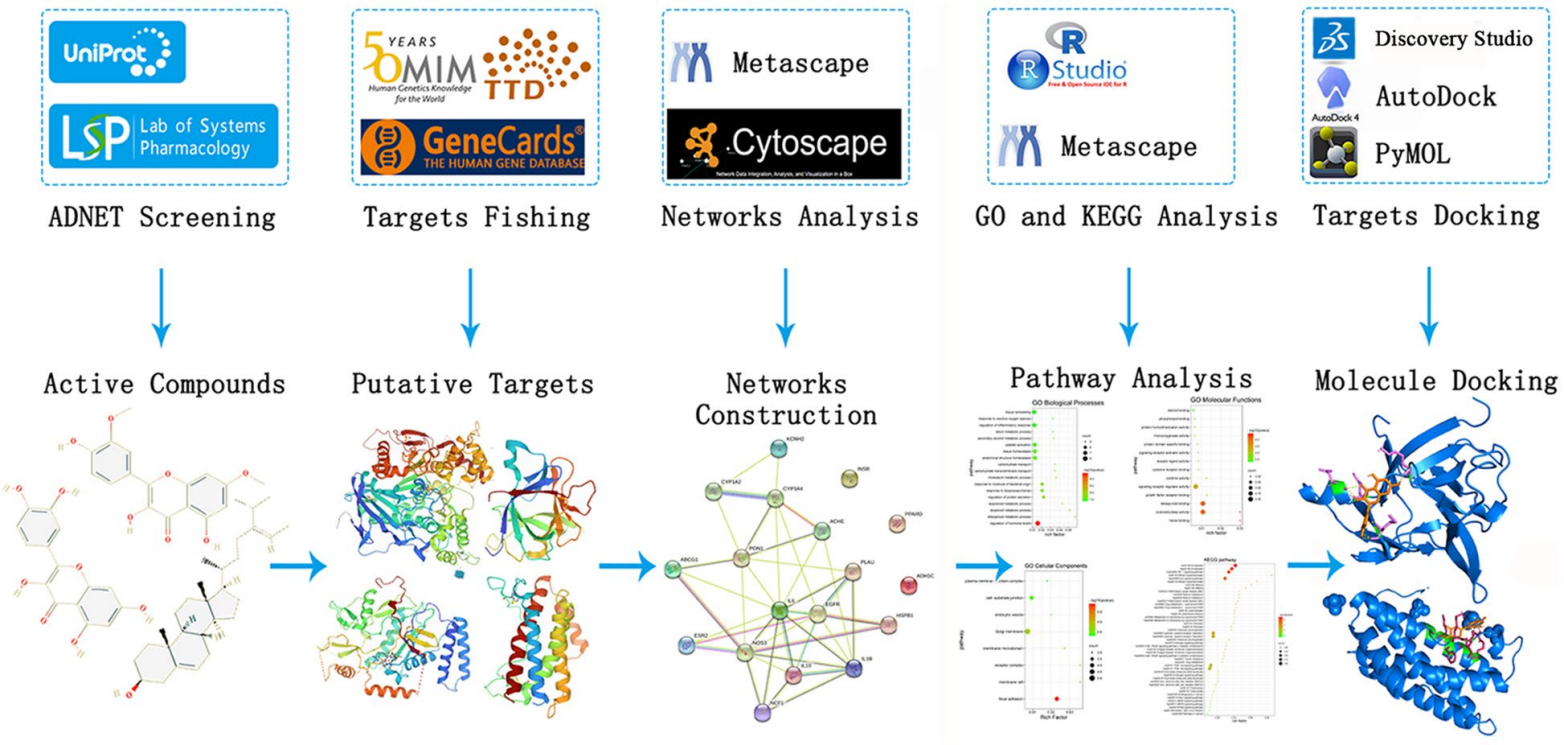
Network pharmacology for identifying mechanisms of *Gynostemma pentaphyllum* (Thunb.) Makino (GpM) in treating Alzheimer’s disease

**Fig. 2:**
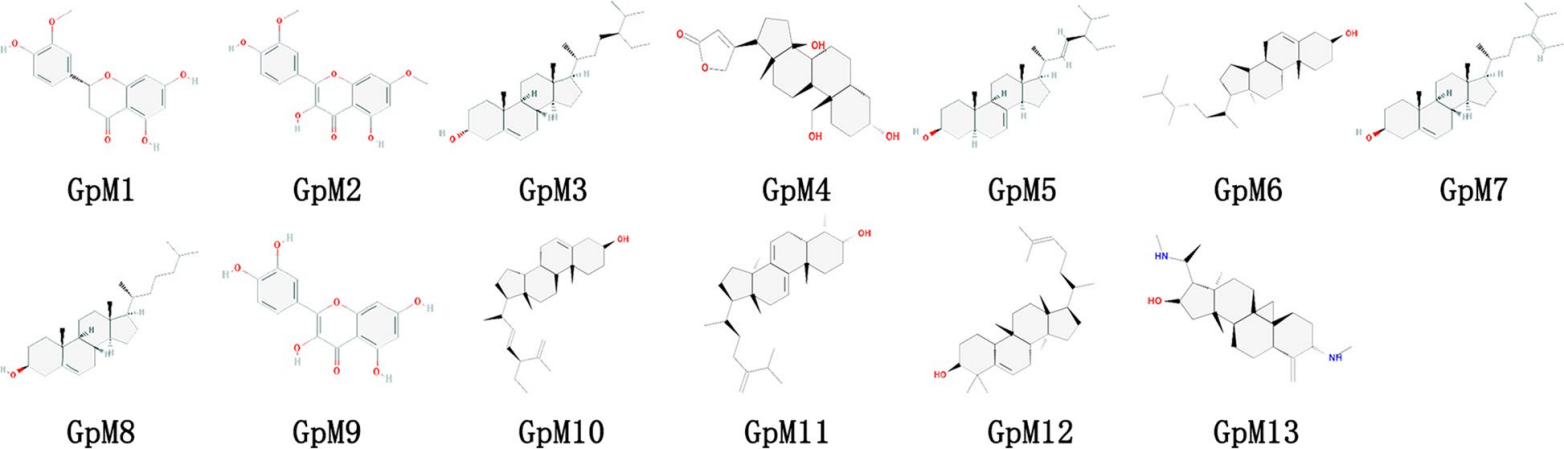
The two-dimensional (2D) molecular structures of 13 active compounds of GpM

We used system pharmacology to find potential pharmacological mechanisms, and each step in defining the role of GpM on AD is shown in Fig. 1. Based on the TCMSP database, 13 compounds were selected as active compounds from GpM (Table-S1), as shown in Fig. 2.

**Fig. 3:**
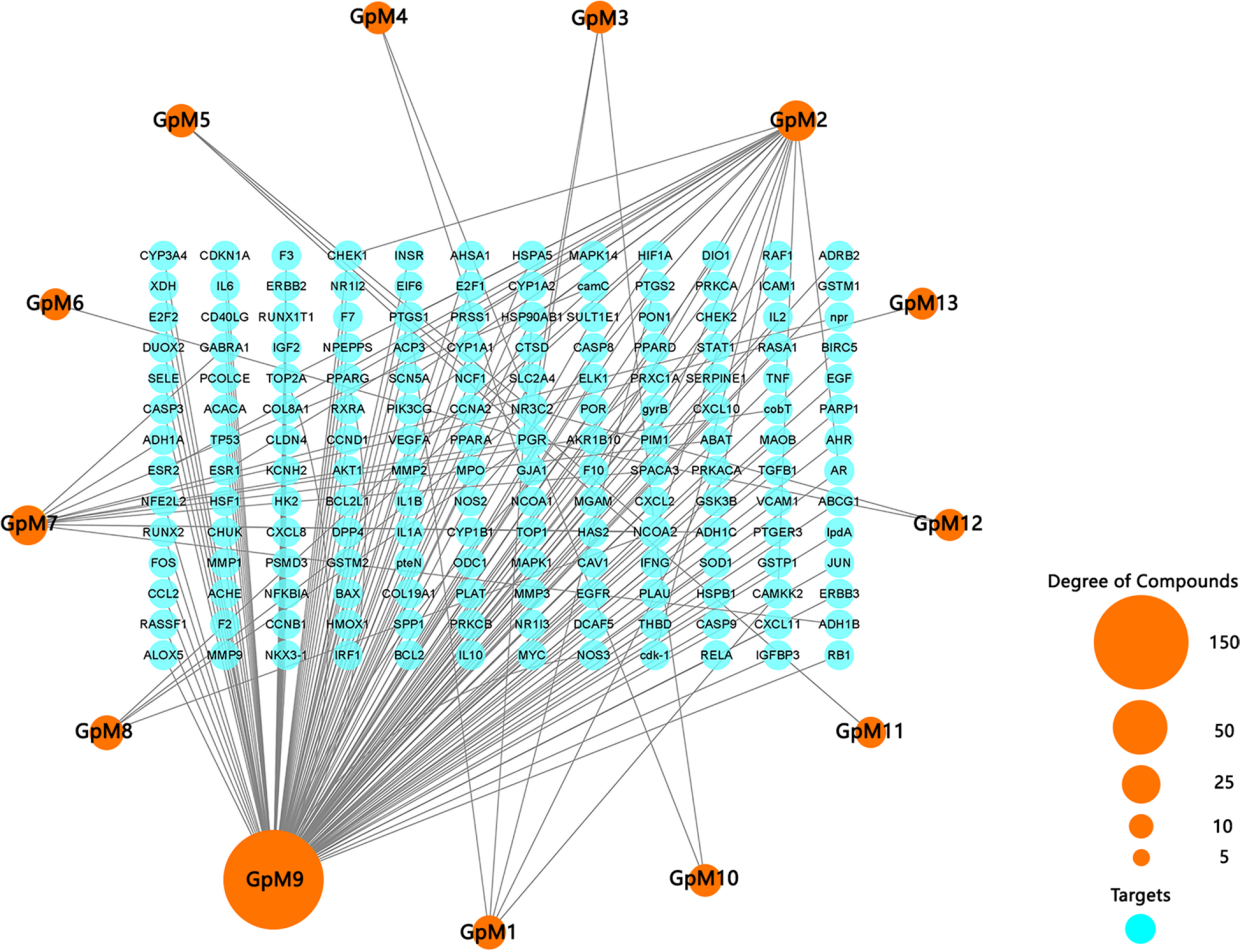
The construction of Compound-Target network. Different active compounds (the circular nodes with orange) were presented in circle. The size of the circular node represents the targets number of compounds. The putative target proteins (the circular nodes with blue) were listed inside the circle made by active compounds

The putative target proteins were screened by docking and binding scores. A total of 168 putative target proteins along with the 13 active compounds were captured by using TCMSP (Table-S2). The value of score represents the relationship between the active compound and the target protein. The higher the score is, the closer the relationship is. The 13 active compounds, 168 putative target proteins and their relationships were shown in Fig. 3.

### AD-associated targets and the intersecting targets between GpM and AD

**Fig. 4:**
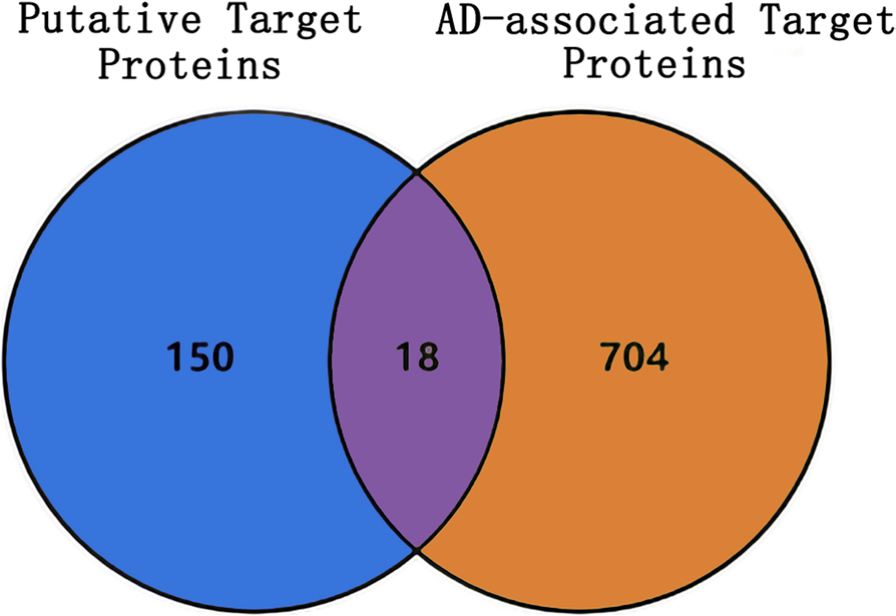
The Venn diagram. The Venn diagram showed the overlap of putative target proteins of GpM and AD-associated targets

We searched three databases and obtained 722 genes entries as AD-associated targets. Then, 18 overlap targets between 168 putative target proteins of GpM and AD-associated targets were regarded as intersecting targets (Fig. 4), including ATP-binding cassette sub-family G member 1 (ABCG1), acetylcholinesterase (ACHE), alcohol dehydrogenase 1C (ADH1C), cytochrome P450 1A2 (CYP1A2), cytochrome P450 3A4 (CYP3A4), epidermal growth factor receptor (EGFR), estrogen receptor beta (ESR2), heat shock protein beta-1 (HSPB1), Interleukin-10 (IL10), interleukin-1 beta (IL-1β), interleukin-6 (IL-6), Insulin receptor (INSR), potassium voltage-gated channel subfamily H member 2 (KCNH2), neutrophil cytosol factor 1 (NCF1), nitric oxide synthase, endothelial (NOS3), urokinase-type plasminogen activator (PLAU), serum paraoxonase/arylesterase 1 (PON1), peroxisome proliferator-activated receptor delta (PPARD), listed in Table-S3.

By screening the active components corresponding to the intersecting targets, three compounds, rhamnazin (GpM2), isofucosterol (GpM7) and quercetin (GpM9), were selected to be as the main compounds.

### Enrichment analysis of intersecting targets

**Fig. 5:**
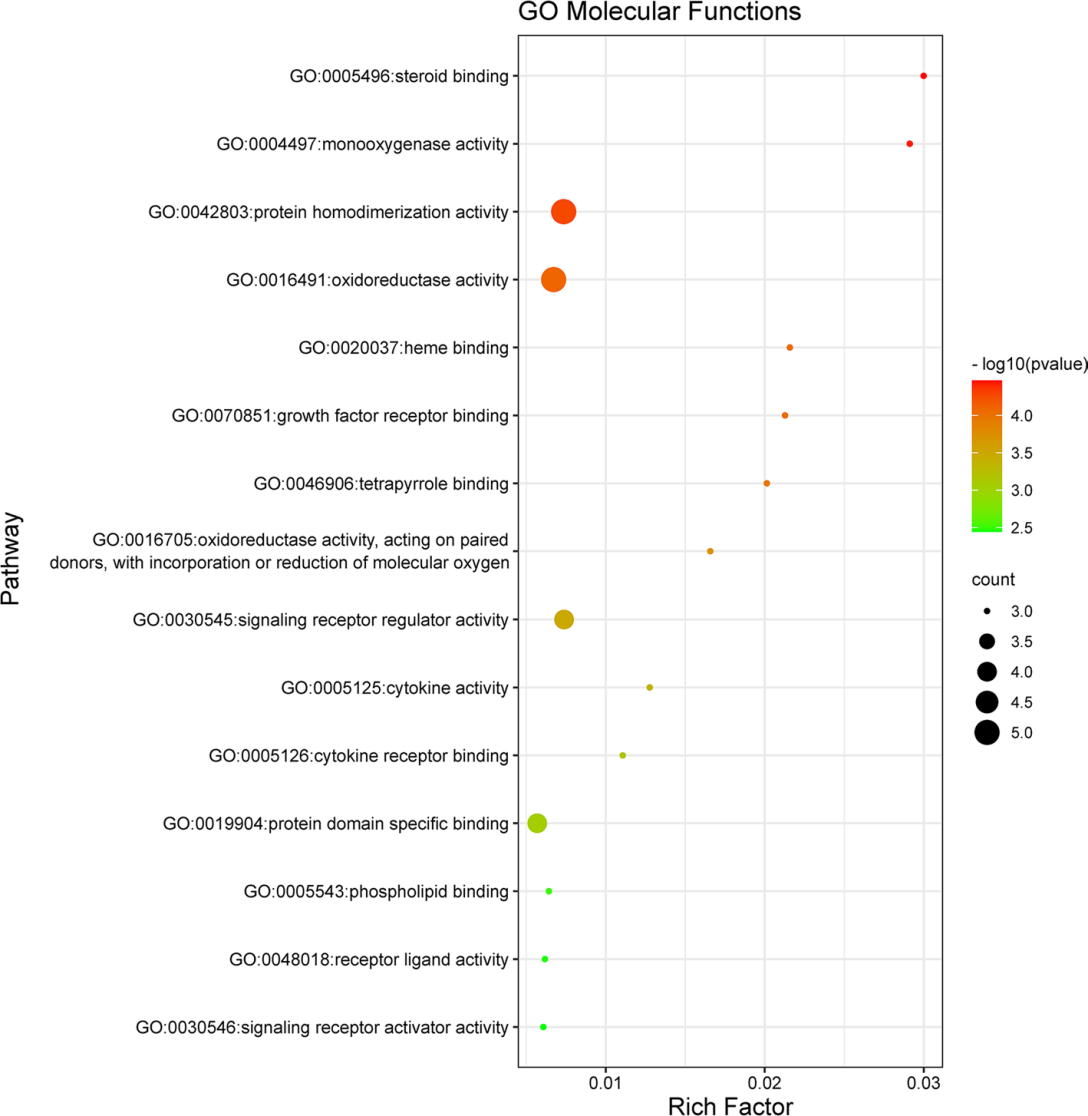
The molecular functions of Gene Ontology (GO) analysis. The significantly enrichment of molecular functions (MF) with *P* < 0.01

**Fig. 6:**
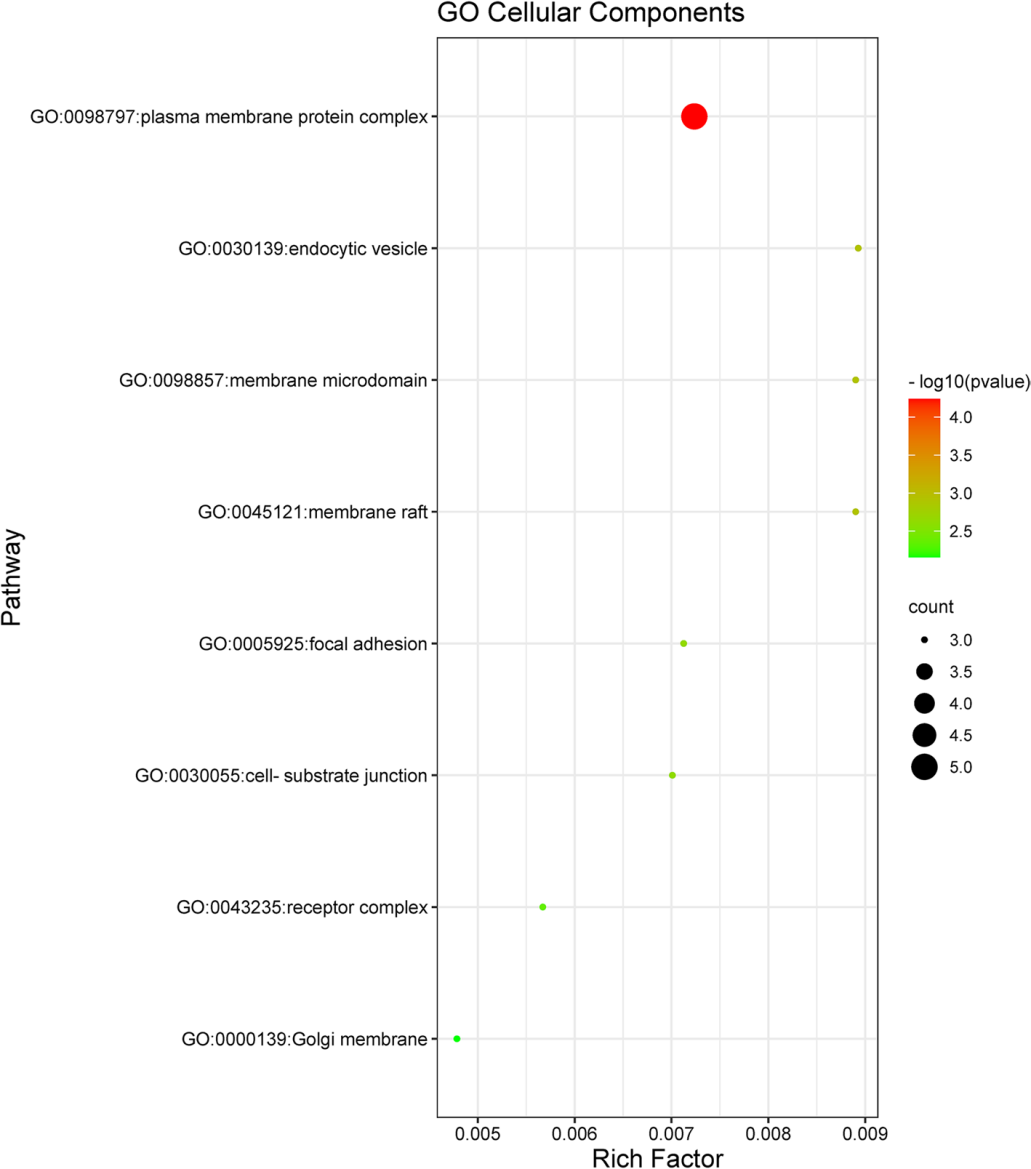
The cellar components of Gene Ontology (GO) analysis. The significantly enrichment of cellar components (CC) with *P* < 0.01

**Fig. 7:**
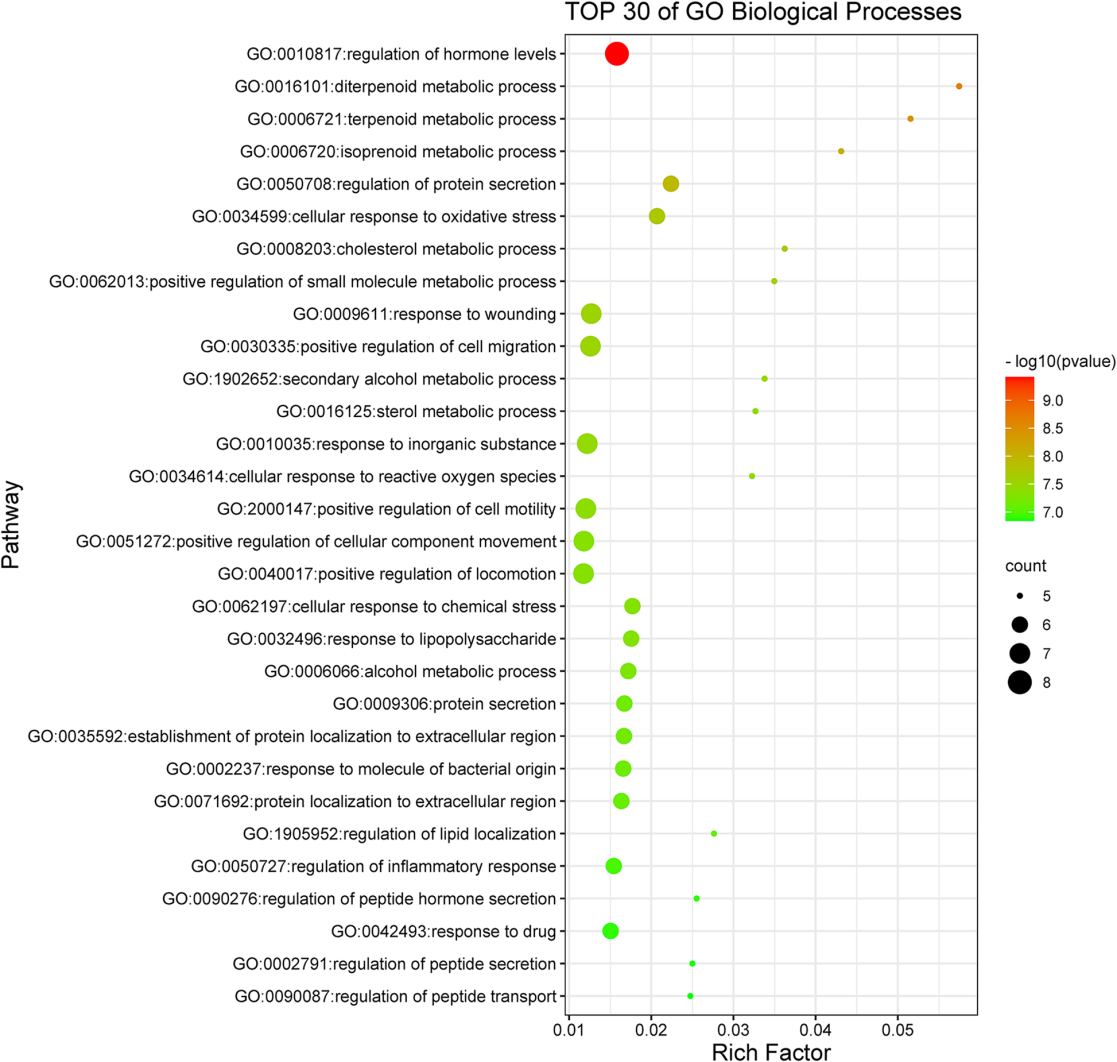
The biological processes of Gene Ontology (GO) analysis. The significantly enrichment of biological processes (BP) (Top 30) with *P* < 0.01

**Fig. 8:**
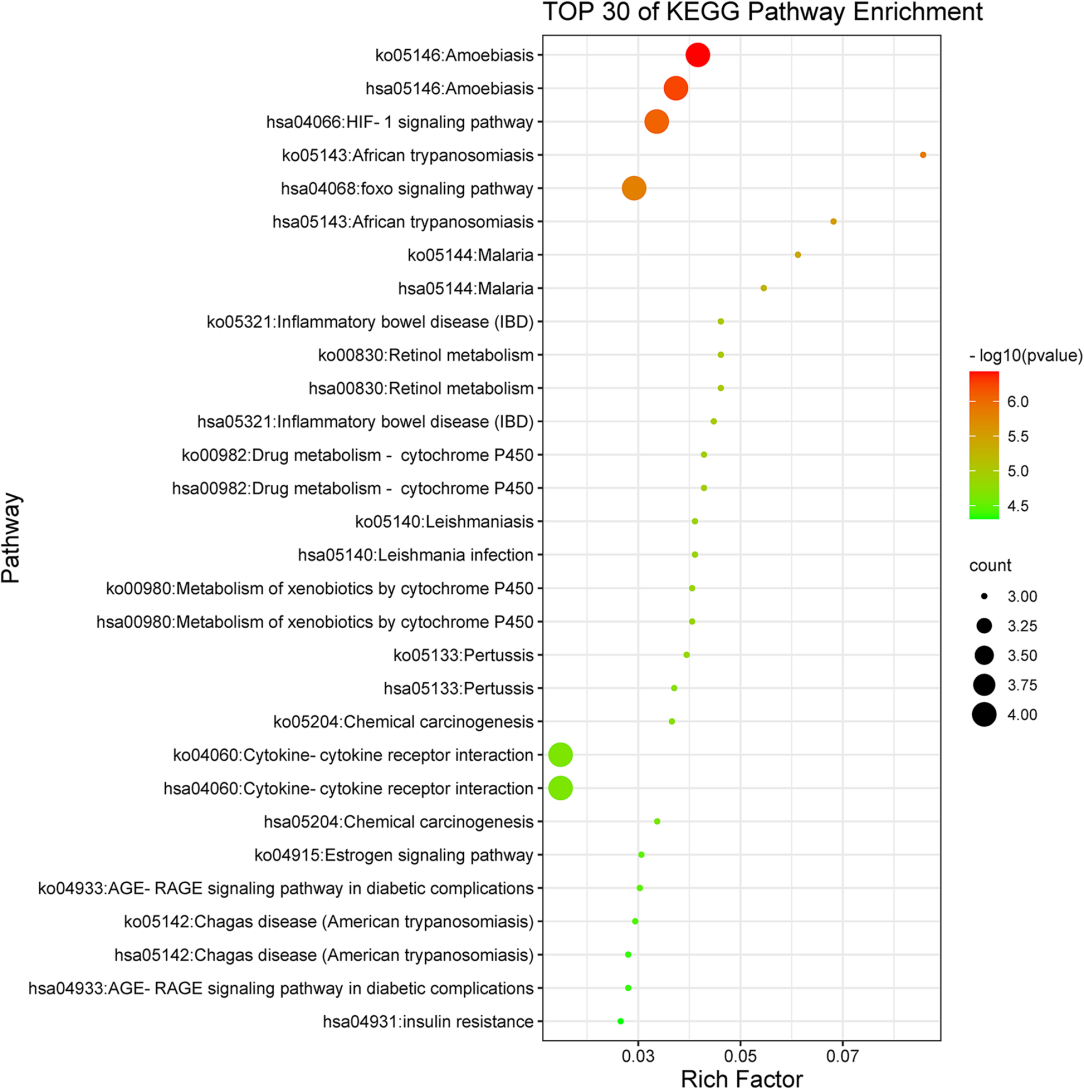
Kyoto Encyclopedia of Genes and Genomes (KEGG) pathway enrichment analysis. The KEGG pathway enrichment analysis with *P* < 0.01 (Top 30) is showed in Fig. 8

GO and KEGG enrichment analyses were performed to identify biological functions and metabolic pathways associated with intersecting targets. Filtering with *P* value < 0.01, a minimum count of 3, 337 GO entries and 47 KEGG pathway entries were identified (Table-S4, Table-S5). Of the 337 GO entries, there were 314 for biological processes, 8 for cellular components, and 15 for molecular function. Next, we used bubble diagram to visually display the results of GO analysis and KEGG pathway enrichment analysis (Figs. 5, 6, 7 and 8).

As shown in Fig. 5, the results showed that the 15 molecular functions of the intersecting targets of GpM and AD mainly concentrated in steroid binding (GO:0005496), monooxygenase activity (GO:0004497), protein homodimerization activity (GO:0042803), oxidoreductase activity (GO:0016491), heme binding (GO:0020037), growth factor receptor binding (GO:0070851), tetrapyrrole binding (GO:0046906), oxidoreductase activity, acting on paired donors, with incorporation or reduction of molecular oxygen (GO:0016705), signaling receptor regulator activity (GO:0030545), cytokine activity (GO:0005125), cytokine receptor binding (GO:0005126), protein domain specific binding (GO:0019904), phospholipid binding (GO:0005543), receptor ligand activity (GO:0048018) and signaling receptor activator activity (GO:0030546).

As shown in Fig. 6, the 8 cellular components of the intersecting targets of GpM and AD were mainly involved in plasma membrane protein complex (GO:0098797), endocytic vesicle (GO:0030139), membrane raft (GO:0045121), membrane microdomain (GO:0098857), focal adhesion (GO:0005925), cell-substrate junction (GO:0030055), receptor complex (GO:0043235) and Golgi membrane (GO:0000139).

As represented in Fig. 7, the top 30 biological processes from the intersecting targets of GpM and AD were principally linked to regulation of hormone levels (GO:0010817), diterpenoid metabolic process (GO:0016101), terpenoid metabolic process (GO:0006721), isoprenoid metabolic process (GO:0006720), regulation of protein secretion (GO:0050708), cellular response to oxidative stress (GO:0034599), cholesterol metabolic process (GO:0008203), positive regulation of small molecule metabolic process (GO:0062013), response to wounding (GO:0009611), positive regulation of cell migration (GO:0030335), secondary alcohol metabolic process (GO:1902652), sterol metabolic process (GO:0016125), response to inorganic substance (GO:0010035), cellular response to reactive oxygen species (GO:0034614), positive regulation of cell motility (GO:2000147), positive regulation of cellular component movement (GO:0051272), positive regulation of locomotion (GO:0040017), cellular response to chemical stress (GO:0062197), response to lipopolysaccharide (GO:0032496), alcohol metabolic process (GO:0006066), protein secretion (GO:0009306), establishment of protein localization to extracellular region (GO:0035592), response to molecule of bacterial origin (GO:0002237), protein localization to extracellular region (GO:0071692), regulation of lipid localization (GO:1905952), regulation of inflammatory response (GO:0050727), regulation of peptide hormone secretion (GO:0090276), response to drug (GO:0042493), regulation of peptide secretion (GO:0002791) and regulation of peptide transport (GO:0090087).

As shown in Fig. 8, the top 30 KEGG signaling pathways of the intersecting targets were Amoebiasis (ko05146, hsa05146), HIF-1 signaling pathway (hsa04066), foxo signaling pathway (hsa04068), African trypanosomiasis (ko05143, hsa05143), Malaria (ko05144, hsa05144), Inflammatory bowel disease (IBD) (ko05321, hsa05321), Retinol metabolism (hsa00830, ko00830), Drug metabolism - cytochrome P450 (hsa00982, ko00982), Leishmania infection (hsa05140, ko05140), Metabolism of xenobiotics by cytochrome P450 (hsa00980, ko00980), Pertussis (ko05133, hsa05133), Chemical carcinogenesis (ko05204), Cytokine-cytokine receptor interaction (hsa04060, ko04060), Chemical carcinogenesis (hsa05204), Estrogen signaling pathway (ko04915), AGE-RAGE signaling pathway in diabetic complications (ko04933, hsa04933), Chagas disease (American trypanosomiasis) (ko05142, hsa05142) and insulin resistance (hsa04931).

### The compound-target-pathway (CTP) and protein-protein interaction (PPI) networks of the intersecting targets

**Fig. 9:**
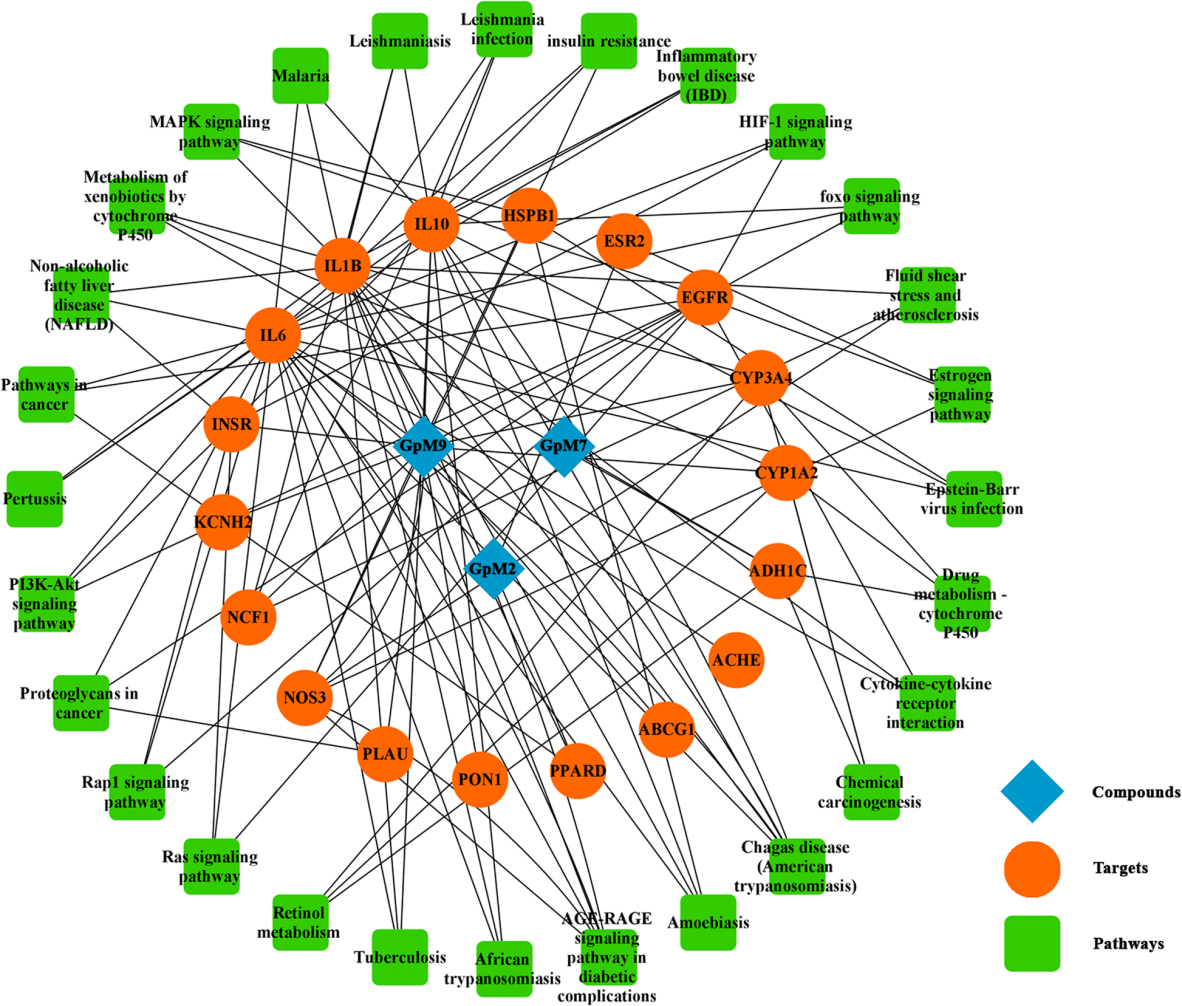
The compound-target-pathway (CTP) network. The 3 active compounds were presented by blue diamond nodes in the center. The target proteins (orange round nodes) which are associated with AD were situated as a round enclosing the 3 compounds. The pathways were listed at the outermost and presented by green round rectangles

**Fig. 10:**
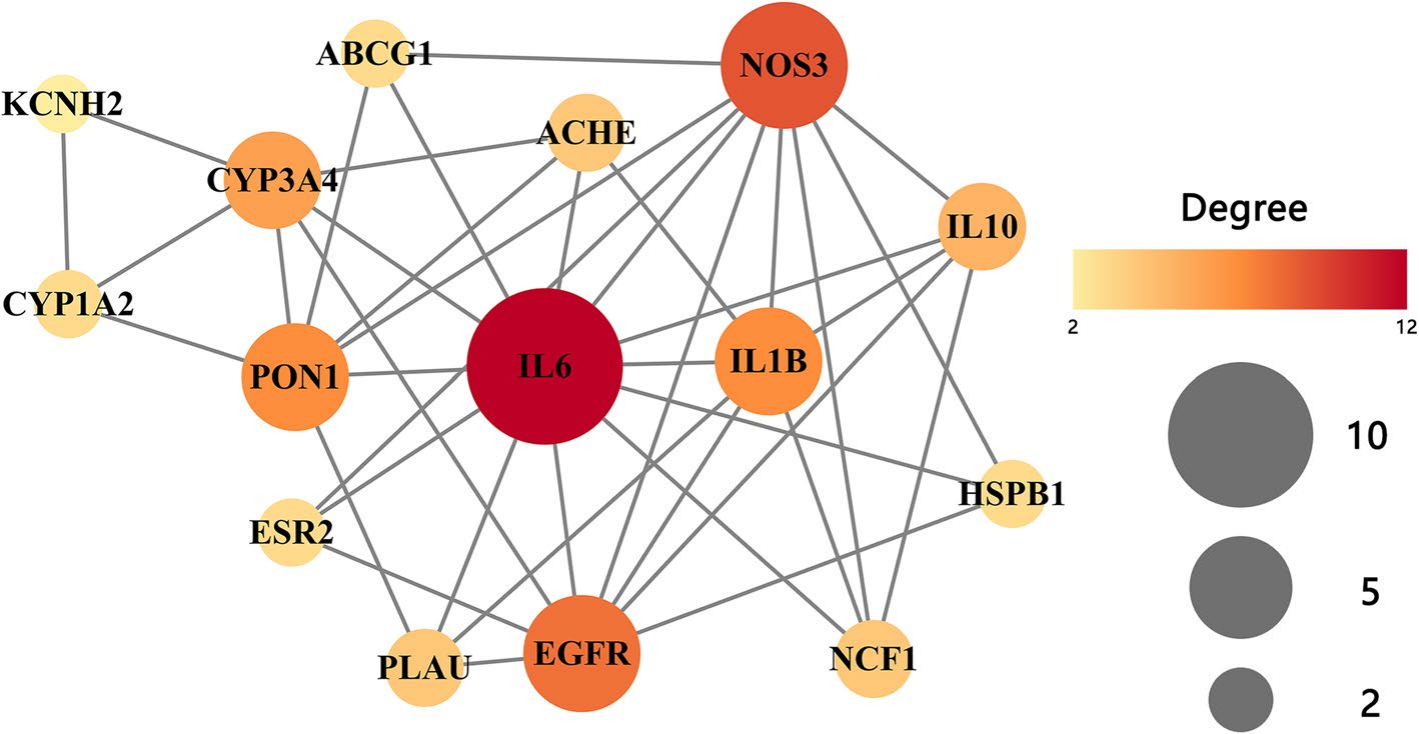
The Protein-Protein Interaction (PPI) network. The top 15 intersecting target proteins were shown in circular nodes with different gradations of red. The gradation of red and size of the nodes expressed the interaction degree on the network

The compound-target-pathway (CTP) and protein–protein interaction (PPI) networks were performed to demonstrate the potential mechanism of GpM on AD. The CTP network showed that 18 proteins and 28 related KEGG pathways (after deleting redundancy) (Fig. 9). The PPI network demonstrated the interaction among the 15 intersecting target proteins (three stray nodes were hidden) (Fig. 10). According to the degree, epidermal growth factor receptor (EGFR), interleukin-1 beta (IL-1β), interleukin-6 (IL-6), nitric oxide synthase, endothelial (NOS3), serum paraoxonase/arylesterase 1 (PON1) were selected as the core target proteins to determine binding energy by means of a molecular docking analysis.

### Molecular docking

**Fig. 11:**
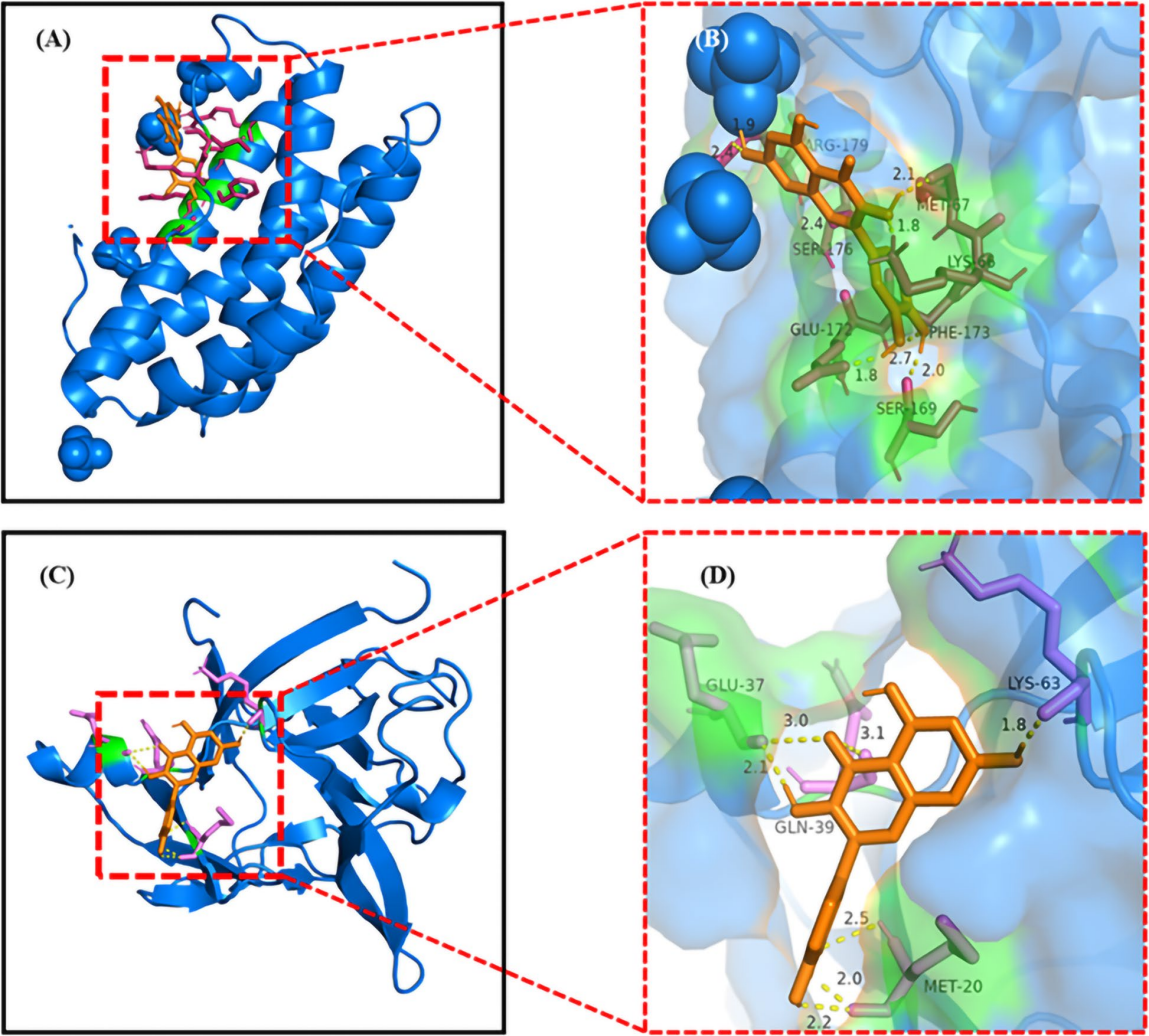
The molecular docking diagrams of quercetin and IL-6, IL-1β using AutoDockTools. The active sites of the proteins, the binding distances, molecular docking model between the proteins and the quercetin were presented in Fig. 11. (**A**, **B**) quercetin and the protein IL-6 (− 7.14 kcal/mol); (**C**), (**D**) quercetin and the protein IL-1β (− 6.21 kcal/mol)

**Fig. 12:**
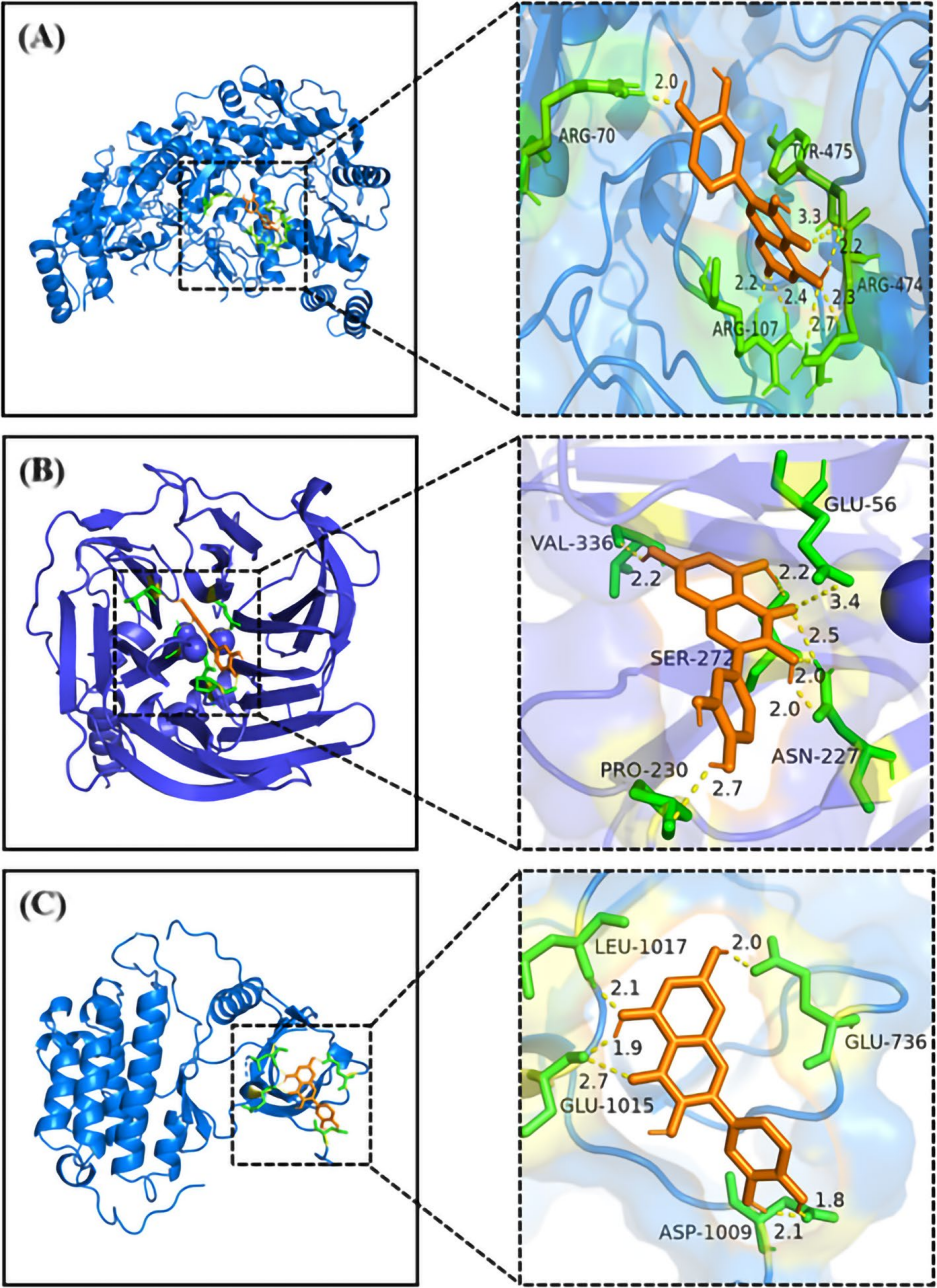
The molecular docking diagrams of quercetin and NOS3, PON1, EGFR using AutoDockTools. The active sites of the proteins, the binding distances, molecular docking model between the proteins and the quercetin were presented in Fig. 12. (**A**) quercetin and the protein NOS3 (− 5.59 kcal/mol); (**B**) quercetin and the protein PON1 (− 6.74 kcal/mol); (**C**) quercetin and the protein EGFR (− 5.52 kcal/mol)

To further verifying the function of GpM on AD, we selected quercetin, one of the three compounds, as the significant compound for the all 5 core targets are part of its putative target proteins. With the utilization of AutoDockTools-1.5.6, OpenBabel-2.4.1 and Pymol-2.2.0 software, the interactions between quercetin and the 5 core targets were analyzed. Figures 11 and 12 showed the molecule docking of 5 core targets with quercetin. The binding energy of each protein with quercetin was shown in Table 1. The lower binding energy is, the higher the affinity between quercetin and the target (protein) is. The hydrogen bonds were found between the active site of proteins and quercetin.

**Table 1:** The binding energy of quercetin and 5 targets

Binding energy (kcal/mol)	IL-6	IL-1β	NOS3	PON1	EGFR
quercetin	−7.14	−6.21	−5.59	− 6.74	−5.52

Note: The binding energy < − 5 kcal/mol was considered as high affinity between quercetin and the target [33]

The 3D model of quercetin in the active site of IL-6 was showed in the Ray tracing diagram in Fig. 11A. The binding energy of quercetin with IL-6 was found to be − 7.14 kcal/mol. The type, distance and number of the binding complexes of the active site of IL-6 and quercetin were shown in the enlarged drawing in Fig. 11B. The following atoms, ARG-179, SER-176, GLU-172, PHE-173, LYS-66 SER-169 and MET-67 formed a pocket around quercetin by hydrogen bonding. Similarly, the binding interaction of quercetin and IL-1β was shown in Fig. 11C. The following atoms, GLU-37, GLN-39, MET-20 and LYS-63 were connected to quercetin by hydrogen bonding (Fig. 11D). The other three docking result were shown in Fig. 12.

**Fig. 13:**
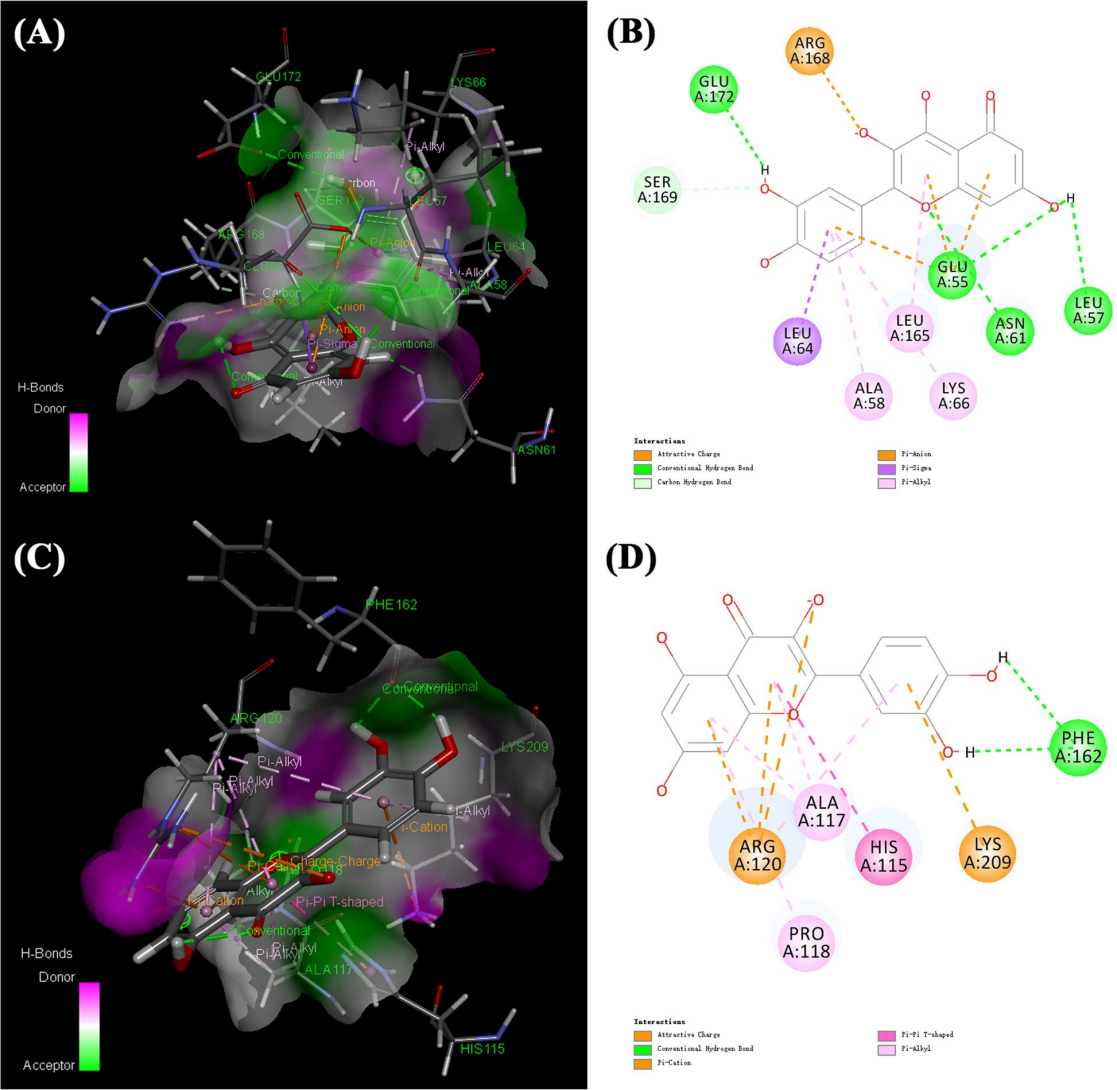
The molecular docking of quercetin and IL-6, IL-1β using Discovery Studio Client. (**A**) the 3D model of quercetin and protein IL-6, (**B**) the 2D model of quercetin and protein IL-6, (**C**) the 3D model of quercetin and protein IL-1β, (**D**) the 2D model of quercetin and protein IL-1β

**Fig. 14:**
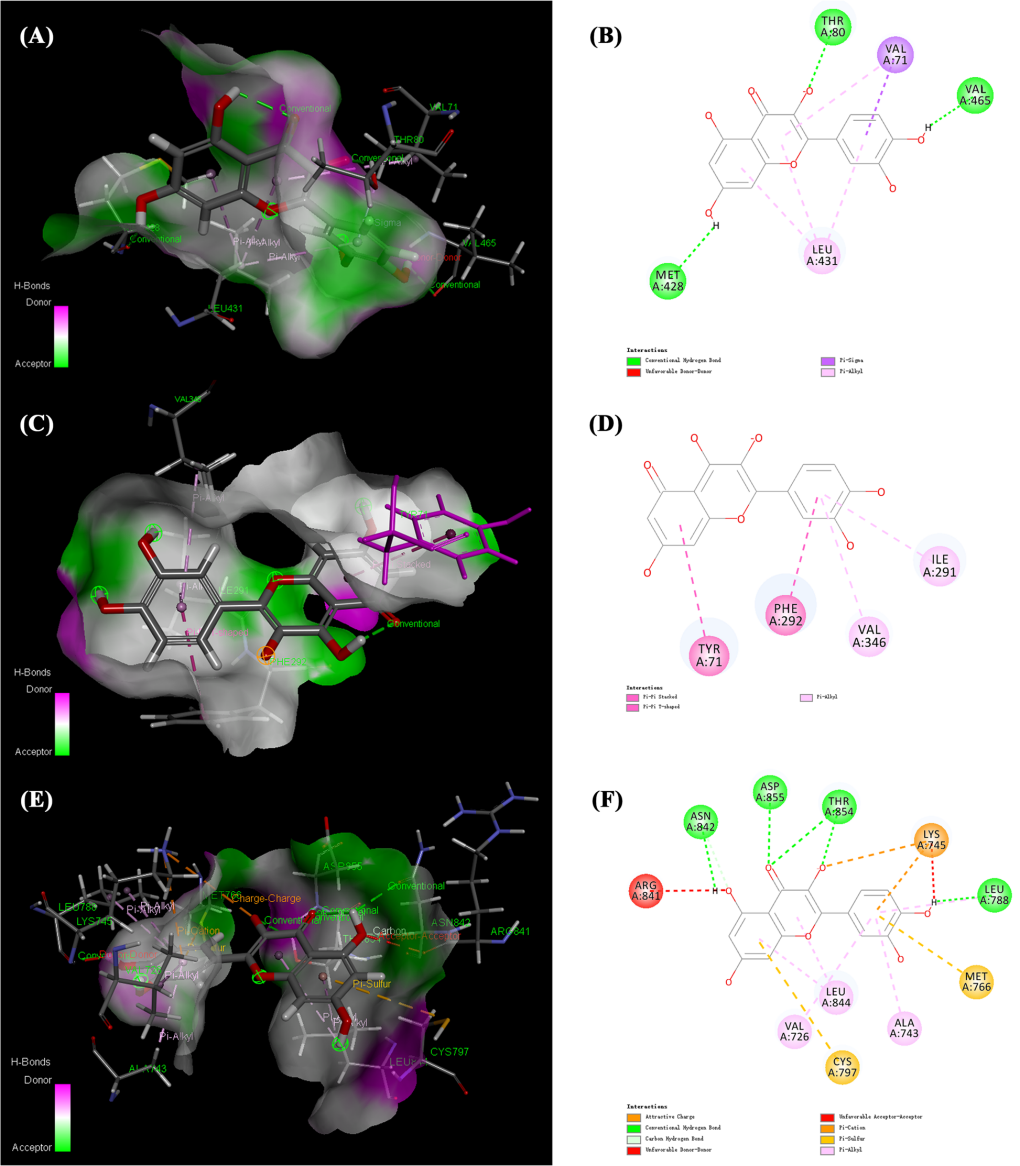
The molecular docking of quercetin and NOS3, PON1, EGFR using Discovery Studio Client. (**A**) the 3D model of quercetin and protein NOS3, (**B**) the 2D model of quercetin and protein NOS3, (**C**) the 3D model of quercetin and protein PON1, (**D**) the 2D model of quercetin and protein PON1, (**E**) the 3D model of quercetin and protein EGFR, (**F**) the 2D model of quercetin and protein EGFR

To further verify the results of molecular docking, we applied Discovery Studio Client to investigate other chemical bonds including Pi-Alkyl, Pi-Sigma and Pi-Pi interaction between quercetin and 5 core targets. The results were shown in Figs. 13 and 14.

Figure 13A showed the 3D model of quercetin and IL-6. To explicitly exhibit the binding environment, 2D model was shown in Fig. 13B. Several bonds were obtained between quercetin and IL-6. There were Pi-Alkyl interactions between quercetin and amino acid residues of IL-6 including LEU-165, LYS-66 and ALA-58. Pi-Sigma interactions exited in active sites including GLU-55 and LEU-64. Figure 13C showed the 3D model of quercetin and IL-1β. The binding environment between quercetin and IL-1β was shown in Fig. 13D. There were Pi-Alkyl interactions between quercetin and IL-1β at the active sites of LYS-209, PRO-118, ALA-117 and ARG-120. In addition, Pi-Pi interactions existed between quercetin and the active site of HIS-115.

The other three docking results were shown in Fig. 14. All the chemical bonds we found with their bond length were shown in Table 2. Multiple interaction sites and chemical bonds indicated that there was great affinity between quercetin and core targets.

**Table 2:** Interaction parameters of core targets with quercetin

Targets	Hydrogen bonding	Pi-Alkyl	Pi-Sigma	Pi-Pi
IL-6	ARG-179(2.4, 1.9) SER-176(2.4) GLU-172(1.8) PHE-173(2.7) LYS-66(1.8) MET-67(2.1) SER-169(2.0)	LEU-165(4.4) LYS-66(5.3) ALA-58(4.6)	GLU-55(3.3, 4.1, 4.9) LEU-64(2.8)	–
IL-1B	GLU-37(3.0, 2.1) GLN-39(3.1) MET-20(2.0, 2.2, 2.5) LYS-63(1.8)	LYS-209(4.9) PRO-118(5.2) ALA-117(3.3, 4.0) ARG-120(3.8, 4.4, 4.8, 5.4)	–	HIS-115(4.2)
NOS3	ARG-70(2.0) TRY-475(2.2, 3.3) ARG-474(2.3, 2.7) ARG-107(2.2, 2.4)	LEU-431(5.2, 4.9, 4.9) VAL-71(5.5)	VAL-71(2.7)	–
PON1	GLU-56(3.4) ASN-227(2.0, 2.0, 2.5) PRO-230(2.7) SER-272(2.2) VAL-336(2.2)	VAL-346(5.2) ILE-291(4.4)	–	PHE-292(4.5) TYR-71(4.9)
EGFR	LEU-1017(2.1) GLU-1015(1.9, 2.7) ASP-1009(1.8, 2.1) GLU-736(2.0)	LEU-844(4.2, 4.9) ALA-743(4.7) VAL-726(5.3) LYS-745(5.0, 4.9) LEU-788(5.3)	–	–

## Discussion

With the development of components analysis of Chinese herbs, TCM shows the characteristics of effective multi-target and multi-component [34]. GpM or some of its components have been shown to possess clinical effects in cardiomyopathy [35], diabetes mellitus [36] and cancer [12]. In recent years, many clinical studies have shown that GpM plays a positive role in alleviating cognitive impairment and brain damage of AD [17, 18]. Although GpM has above medicinal value, the related mechanism remains unclear.

In this study, we explored the active compounds of GpM, and selected the intersecting targets between the compounds and AD. 18 targets from the 168 putative target proteins of compounds and 722 AD-associated targets were obtained. Furthermore, multiple signaling pathways, biological processes, cellular components and molecular functions were identified by GO/KEGG pathway enrichment analysis. It is considered that seven pathways were closely related to the pathogenesis of AD including amoebiasis (ko05146, hsa05146), HIF-1 signaling pathway (hsa04066), African trypanosomiasis (ko05143, hsa05143), foxo signaling pathway (hsa04068), malaria (ko05144, hsa05144), inflammatory bowel disease (IBD) (ko05321), retinol metabolism (ko00830, hsa00830). Five essential proteins (IL-6, IL-1β, NOS3, PON1 and EGFR) were screened out using CTP and PPI network. Due to good molecular docking results presented by Auto Dock Tools, it is theoretically proved they were target proteins for further study of the anti-AD effects of GpM.

Consistent with our results, HIF-1 signaling pathway and foxo signaling pathway are involved in the occurrence and development of AD, which has been reported in previous studies [37, 38]. Inflammatory bowel disease (IBD) is considered to be closely related to pathogenesis of AD, based on studies of gut-brain axis [39]. Additionally, vitamin A deficiency has been demonstrated in patients with AD [40], while vitamin A supplementation can alleviate the development of AD [41]. More interestingly, our study suggests that the AD-associated pathways may relate to amoebiasis, African trypanosomiasis and malaria, which are all caused by parasitic infections and may invade the central nervous system. Existing studies have shown the existence of anti-parasitic infection and anti-AD drugs (such as GSK-3 inhibitors) [42], which imply that the incidence of AD may relate to parasitic infection. To a certain extent, it shows the credibility and innovation of our conclusion.

Previous studies have shown that inflammatory cytokines are involved in AD [43, 44]. A recent study showed that elderly individuals with amyloid-beta deposition had higher levels of IL-1β and IL-6 [45]. In addition, it has been reported that higher level of Aβ42 can reduce endothelial NO synthase (eNOS, NOS3), cyclic GMP (cGMP) and protein kinase G (PKG) activity [46]. This is consistent with our finding that GpM may prevent against AD by regulating the activity of NOS3 and the levels of IL-1β and IL-6. PON1 is a new factor associated with impaired cognition and may play a role in the development of AD [47]. It is reported that EGFR is related to AD, and EGFR inhibitors can be used as burgeoning therapeutic strategy for AD [48]. These data suggest that targeting on IL-1β, IL-6, NOS3, PON1 or EGFR may be effective in the treatment of AD, which supports our finding that proteins with good molecular docking are the important target proteins for neuroprotection of GpM in the treatment of AD.

In conclusion, based on network pharmacology and bioinformatics, this study illustrated the key targets and molecular mechanisms of GpM, which will provide instructive suggestions for the further study of GpM in the treatment of AD.

## Conclusions

In this study, we applied network pharmacology and bioinformatics to analyze the therapeutic effect of GpM on AD. The active components and putative targets of GpM were explored and discussed systematically. Comparing putative targets of GpM with known AD related genes, constructing and analyzing CTP/PPI networks, 5 important proteins were identified, showing strong therapeutic potentials against AD. Through enrichment analysis, we further identified GpM as a promising drug with multiple components, targets and pathways for the treatment of AD. In addition, we demonstrated the feasibility of GpM in the treatment of AD by molecular docking. In conclusion, our findings provide a new idea for the neuroprotection of GpM and contribute to the development of GpM in the treatment of AD. However, data mining and analysis alone is not enough. In future, we will conduct experiments to confirm our points and provide more effective treatment measures for AD patients.

## Supplementary Information

**Additional file 1: Table-S1.** Result of 13 candidate compounds of GpM.

**Additional file 2: Table-S2.** Result of 168 putative target proteins of GpM.

**Additional file 3: Table-S3.** 722 AD-associated target proteins of *Homo sapiens* from OMIM, TTD and Gene Cards were regarded as the substantially contributing proteins.

**Additional file 4: Table-S4:** Result of 337 GO terms enrichment of GpM3.

**Additional file 5: Table-S5.** Result of the KEGG pathway enrichment analysis of GpM.

## Data Availability

The datasets used and/or analyzed during the current study available from the corresponding author on reasonable request.

## Abbreviations

AD: Alzheimer’s disease
GpM: Gynostemma Pentaphyllum (Thunb.) Makino
GO: Gene Ontology
KEGG: Kyoto Encyclopedia of Genes and Genomes pathway enrichments
CTP: compound-target-pathway
PPI: protein–protein interaction
HIF-1: hypoxia inducible factor-1
EGFR: epidermal growth factor receptor
IL-1β: interleukin-1 beta
IL-6: interleukin-6
NOS3: nitric oxide synthase in endothelial
PON1: serum paraoxonase/arylesterase 1
Aβ: amyloid beta
NFTs: neurofibrillary tangles
ABCA7: ATP-binding cassette transporter A7
BIN1: Bridging integrator 1
CASS4: Cas scaffolding protein family member 4
CD33: Myeloid cell surface antigen CD33
CD2AP: CD2-associated protein
CELF1: CUGBP Elav-like family member 1
CLU: Clusterin
CR1: Complement receptor type 1
DSG2: Desmoglein-2
TCMSP: Traditional Chinese Medicine Database and Analysis Platform
ABCG1: ATP-binding cassette sub-family G member 1
ACHE: acetylcholinesterase
ADH1C: alcohol dehydrogenase 1C
CYP1A2: cytochrome P450 1A2
CYP3A4: cytochrome P450 3A4
ESR2: estrogen receptor beta
HSPB1: heat shock protein beta-1
IL10: Interleukin-10
INSR: Insulin receptor
KCNH2: potassium voltage-gated channel subfamily H member 2
NCF1: neutrophil cytosol factor 1
PLAU: urokinase-type plasminogen activator
PPARD: peroxisome proliferator-activated receptor delta
TCM: Traditional Chinese Medicine
eNOS: endothelial NO synthase
cGMP: cyclic GMP
PKG: protein kinase G
OB: oral bioavailability
DL: drug-likeness
